# The Prevalence of Multi-Type Infections Among Human Papillomavirus Types in Korean Women

**DOI:** 10.3390/pathogens14040369

**Published:** 2025-04-09

**Authors:** Jang Mook Kim, Hee Seung Song, Jieun Hwang, Jae Kyung Kim

**Affiliations:** 1 Department of Health Administration, College of Health Science, Dankook University, Cheonan 31116, Republic of Korea; 2 Department of Nursing, Sangji University, Wonju 26339, Republic of Korea; 3 Department of Biomedical Laboratory Science, College of Health Science, Dankook University, Cheonan 31116, Republic of Korea

**Keywords:** human papillomavirus, infections, genotypes, cervical screening

## Abstract

The distribution of human papillomavirus (HPV) genotypes shows inconsistencies across countries, ethnicities, and socioeconomic levels. An in-depth identification of HPV infection rates and genotypes across regions, ethnicities, and age groups in large populations is crucial. We aimed to assess the prevalence of HPV infections among Korean women and investigate the prevalence of multi-type infections among HPV types. To identify HPV types, 16,669 specimens were subjected to DNA extraction and real-time polymerase chain reactions. The HPV infection rate was 36.7%, with single- and multi-type HPV infection rates of 21.4% and 15.3%, respectively. The prevalence of HPV infection was higher among women in their 20s and 60s. HPV types 16 and 18 were most commonly multi-type infected with HPV type 52. In conclusion, promoting HPV awareness and prevention strategies and incentivizing vaccination can boost vaccination rates among eligible individuals.

## 1. Introduction

Human papillomavirus (HPV) is a common sexually transmitted infection [1]. In 2022, an estimated 599,130 new cases of cervical cancer were reported among women of all ages worldwide, resulting in 315,360 deaths attributed to the disease [2]. HPV infection, especially persistent high-risk (HR) HPV infection, has been identified as the leading cause of cervical cancer [3,4]. HPV infections can lead to health problems and increased healthcare costs [5].

To date, more than 200 different HPV types have been identified in various epidemiological studies [6]. The International Agency for Research on Cancer lists 12 HPV genotypes (HPV 16, 18, 31, 33, 35, 39, 45, 51, 52, 56, 58, and 59) as class 1 carcinogens. HPV types are categorized into high- and low-risk viruses based on whether they cause cancer. HPV infections are categorized into single- or multi-type infections (infections with two or more types of viruses), with HR-HPV multi-type infections increasing in recent years [7].

The clinical implications of multi-type HPV infections remain controversial [8]. Although some studies have suggested that multi-type infections are associated with a higher risk of cervical lesions [9,10,11], others have reported no significant difference in risk compared to single-type HR-HPV infections [12,13,14]. The interactions between co-infecting HPV genotypes—whether synergistic or competitive—are still being elucidated [15,16].

Complicating this landscape is the observation that HPV DNA is not always detectable in advanced cervical lesions. Some studies have proposed that immune clearance or viral integration into the host genome may lead to viral disappearance in later disease stages [17,18]. A possible explanation for this phenomenon is the “hit-and-run” mechanism, wherein HPV initiates oncogenic transformation but is no longer present as the lesion progresses [17]. Furthermore, the prevalence and distribution of HPV genotypes vary by region, ethnicity, and socioeconomic status [19,20], adding further complexity. Therefore, the continuous surveillance of HPV infection patterns across demographic groups is essential. In particular, age- and genotype-specific epidemiological data can support evidence-based vaccination strategies and help to develop more effective public health interventions [21].

In this study, we aimed to evaluate the prevalence of HPV infections among Korean women. We specifically focused on identifying multi-type infections involving HPV 16 and 18, which together account for approximately 70% of cervical cancer cases [22]. Our findings may offer valuable insights for the development and refinement of HPV prevention and screening programs tailored to the Korean population.

## 2. Materials and Methods

### 2.1. Study Design and Methods

**Figure 1:**
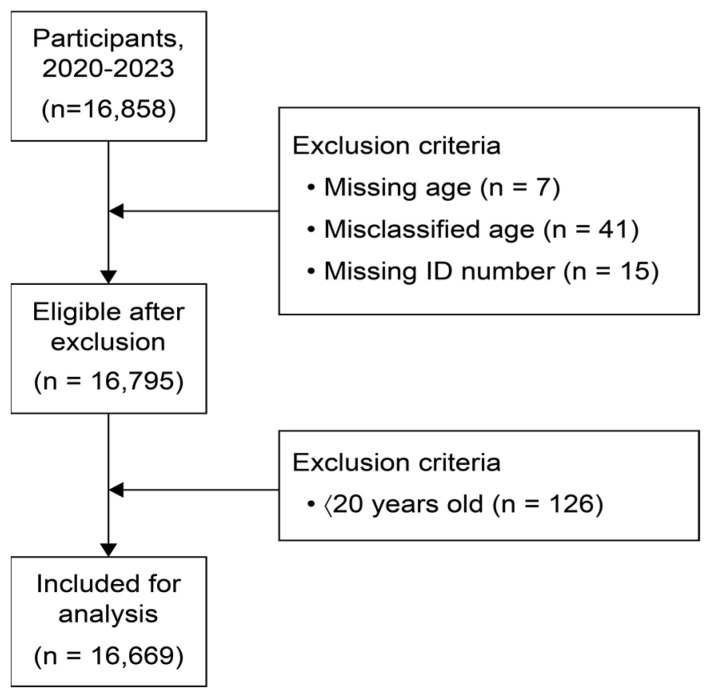
The sample selection process for study participants.

This cross-sectional study was conducted over a 30-month period from January 2020 to June 2023. In this study, we analyzed only the results of specimens that were requested for HPV testing from hospitals across the country to the outsourced testing center (U2Bio). The sample collection procedure is illustrated in Figure 1. A total of 16,669 samples from the general female population were included in this study. Samples from individuals under 20 years were excluded due to differences in immune responses between adult and teenage populations, as well as the objectives of this study. No personally identifiable information was collected. All samples were processed at a single testing institution using the standardized nucleic acid extraction protocol and polymerase chain reaction (PCR) systems. After specimen collection, DNA extraction and multiplex real-time PCR (RT-PCR) were performed to identify HPV genotypes. All testing procedures were conducted following the protocols of the respective manufacturers.

### 2.2. DNA Extraction and RT-PCR Amplification

Swabs, tissues, urine, and other samples were collected. Cervical samples were collected using a sterile swab by rubbing the cervix. Tissue samples were collected through biopsy, and urine samples were collected once. QIAsymphony (QIAGEN, Hilden, Germany) was used to extract DNA from the collected samples. RT-PCR was performed using the OmniPlex-HPV™ kit (Genematrix, Seongnam, Republic of Korea) and a CFX96 real-time thermocycler (Bio-Rad, Hercules, CA, USA). Forty-one genotypes were detected, including 12 HR (16, 18, 31, 33, 35, 39, 45, 51, 52, 56, 58, and 59) and 29 low-risk (6, 11, 26, 30, 32, 40, 42, 43, 44, 53, 54, 55, 61, 62, 66, 67, 68, 69, 70, 71, 72, 73, 74, 81, 82, 83, 84, 87, and 99) types. In this study, HPV positivity was defined as a positive result for one or more of the 41 HPV genotypes, regardless of the specific genotype detected.

### 2.3. Data Analysis

All statistical analyses were conducted using SAS software (version 9.4, SAS Institute, Cary, NC, USA). For continuous data, the mean and standard deviation were calculated. For nominal data, frequencies and percentages are presented as descriptive statistics, and differences between variables were tested using the chi-square test. Statistical significance was set at *p* values < 0.05.

## 3. Results

### HPV Infection Rate

The HPV infection rate among 16,669 women was 36.7% (Table 1). The single- and multi-type infection rates were 21.4% and 15.3%, respectively. The HPV positivity rates by age group were highest among women in their 20s at 56.6%, followed by those in their 60s at 40.5%, 70s and older at 36.7%, 30s at 35.4%, 50s at 32.9%, and 40s at 28.3%. The difference in positivity rates across the age groups was statistically significant (*p* < 0.0001).

A comparison of the single- and multi-type infections by age group showed that the single-type infection rates were highest in women in their 20s (26.1%), followed by those in their 60s (23.6%), 50s (21.0%), and 30s (20.8%). Similarly, multi-type infections were highest in women in their 20s (30.5%), followed by those in their 70s and older (17.1%), 60s (16.8%), and 30s (14.5%). The difference in multi-type infection rates across age groups was statistically significant (*p* < 0.0001). The mean age of individuals with HPV, single-type infections, and multi-type infections was 42.9 ± 14.1, 44.1 ± 13.3, and 41.4 ± 14.9 years, respectively.

Among the 16,669 women, the prevalence of HPV single-type infections was 21.4%, followed by 8.6% for multi-type infections with double-type viruses, 3.8% for multi-type infections with triple-type viruses, and 2.9% for multi-type infections with quadruple-type viruses or greater (Table 2). Regardless of the number of multi-type infection viruses, women in their 20s had the highest infection rate, followed by those in their 60s for single-, double-, and triple-type viruses, as well as those in their 70s for quadruple-type viruses or greater. Significant differences in infection rates by the number of HPV types were observed across age groups (*p* < 0.0001).

**Figure 2:**
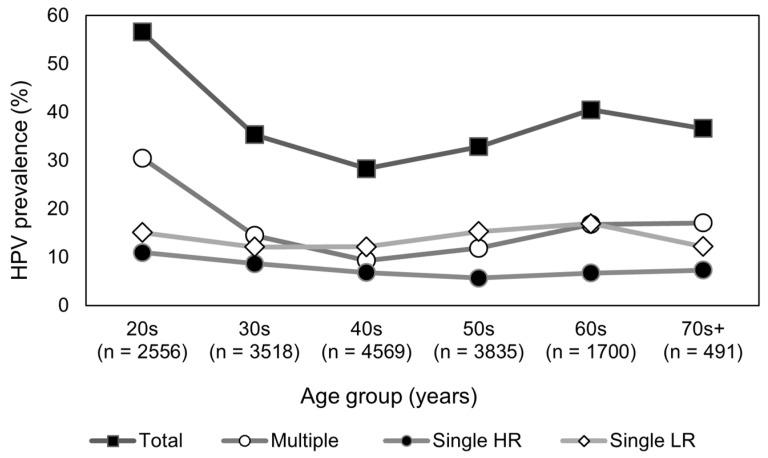
Age-specific positivity rates for total HPV (Total), single-type HR-HPV (Single HR), single-type LR-HPV (Single LR), and multi-type HPV (Multiple) types. HPV, human papillomavirus; HR-HPV, high-risk human papillomavirus; LR-HPV, low-risk human papillomavirus.

Regarding the overall HPV positivity rate, single-type HR-HPV positivity rate, single-type low-risk HPV positivity rate, and HPV multi-type infections by age, the overall HPV positivity rate was highest in women in their 20s and lowest in those in their 40s, which then increased in those in their 50s and 60s (Figure 2).

The single HR-HPV positivity rate was highest in women in their 20s, decreased in those in their 50s, and increased in those in their 60s. The rate of single low-risk HPV positivity decreased in women in their 30s, increased in those in their 50s, and peaked in those in their 60s. HPV multi-type infection rates were highest in women in their 20s, with the lowest prevalence in those in their 40s, which increased from those in their 50s to those in their 70s.

**Figure 3:**
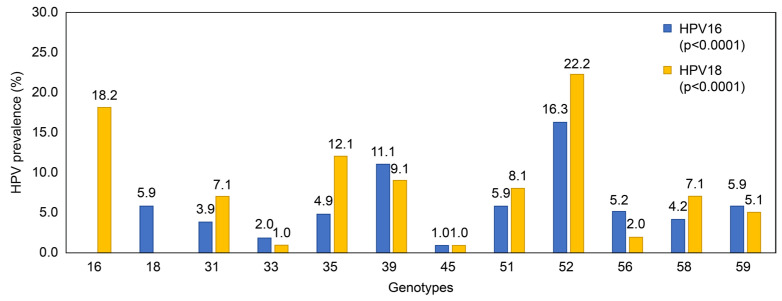
Multi-type infection rates by genotype for HPV 16 and HPV 18.

The assessment of the infection rate by HR genotype among individuals with multi-type infections revealed that 550 women were infected with HPV 16, including 243 and 307 women with single- and multi-type infections, respectively. Among women with multi-type infections, 5.9% had multi-type infections with HPV 18. Among women with HPV 16 multi-type infections, the most common multi-type infection was HPV 52 (16.3%), followed by HPV 39 (11.1%) (Figure 3).

Conversely, 156 women were infected with HPV 18 (Figure 3), including 57 individuals with single-type infections and 99 with multi-type infections. Among individuals with multi-type infections, 18.2% had multi-type infections with HPV 16, and among those with HPV 18 multi-type infections, the most common multi-type infections were HPV 52 (22.2%), HPV 16 (18.2%), and HPV 35 (12.1%). The age group-specific distribution of single- and multi-type HPV infections, including HR and LR classifications, is summarized in Supplementary Table S1.

## 4. Discussion

In this study, we examined HPV positivity rates among 16,669 Korean women in the general population and found that the overall HPV infection rate was 36.7%, with 21.4% for single-type infections and 15.3% for multi-type infections. These rates differed from those reported by Kim et al. [23], who studied 814 women in a Korean hospital who were tested for HPV, particularly in terms of single-type infection rates (reported as 44.3% for 361 cases). The rate of multi-type HPV infections in their study (13.5%; 110 cases) was similar to that in our study, with single-type infections being more common than multi-type infections. The difference in the single-type infection rate might be attributable to the average age, which was 55.2 (±11.9) years in the study by Kim et al. and 44.1 (±13.3) years in the present study. In addition, Kim et al. included women with abnormal cytology and histology results or those undergoing routine checkups. In contrast, we included women who underwent routine checkups and were tested for suspected sexually transmitted infections. The difference in sample sizes between the studies may also have contributed to the difference in single-type infection rates [23].

In this study, single- and multi-type infection rates were higher in younger patients (in their 20s) than those in older age groups. While additional data specific to this study were not collected to substantiate the high HPV infection rates among those in their 20s, higher rates of HPV infection in this age group are commonly reported and are often attributed to several factors, including the number of sexual partners [21,24]. Individuals in their 20s exhibited the highest rates of viral infections, and those in their 60s showed the next highest infection rates.

Individuals in their 20s exhibited the highest rates of single-, double-, triple-, and quadruple-type viral infections; those in their 60s showed the highest rates of single-, double-, and triple-type viral infections; and those in their 70s had the highest rates of quadruple-type viral infections. In addition to the high rate of HPV infection among individuals in their 60s, they also showed a higher number of infected viral types than those in other age groups, except for those in their 20s. The exact mechanism linking the number of infected viral types to the carcinogenic effect remains unclear. HPV may also be associated with other malignancies, including oropharyngeal, anogenital, and other HPV-related tumors. Therefore, comprehensive management and monitoring strategies, including the prevention of HPV infection, remain crucial.

The overall HPV positivity rates peaked among individuals in their 20s, declined among those in their 40s, and then increased again. Similarly, the multiple-type HPV positivity rates peaked in those in their 20s, declined in those in their 40s, and continued to rise for those in their 70s. Thus, both the overall HPV and multiple-type HPV positivity rates followed a U-shaped pattern, with peaks observed in both younger and older age groups, consistent with findings from previous studies [12]. These findings are similar to those of a study by Zhong et al. involving 73,596 Chinese women aged 13–94 years, wherein the authors found that women under 25 and over 65 years were more susceptible to multi-type infections than those in the other age groups studied [25]. The U-shaped pattern is largely attributed to high levels of sexual activity in young people, a decline in immunity, and HPV reactivation in postmenopausal women [26,27,28].

Although we could not distinguish between recent and persistent infections due to additional data collection limitations in this study, previous studies have shown that persistent HR-HPV infection is associated with the development of invasive cervical cancer [29]. Older women, who are more likely to have a higher prevalence of cervical cancer, should be closely monitored, particularly those with HR-HPV infections [30].

In this study, HPV types 16 and 18 were found most frequently in multi-type infections with HPV type 52. These findings are similar to those of Dalstein et al. [29], who examined HPV testing results in 748 female patients at a hospital in South Korea. Additionally, Zheng et al. [31] reported HPV 52 as one of the most common genotypes found in Chinese women, alongside HPV 16.

Moreover, HPV 52 was found to be more prevalent in HPV multi-type infections than in HPV single-type infections [22]. According to data from the Korea Centers for Disease Control and Prevention in 2024, the nationwide coverage rate of bivalent or quadrivalent primary vaccination for girls aged 12–17 years is approximately 77.5% for those born in 2011, supported by government immunization programs [32]. However, HPV type 52 is included in the 9-valent vaccine and is not covered by the Korean National Immunization Program, indicating individuals need to pay for it to receive its benefits.

Previous studies have reported conflicting findings regarding the carcinogenic risk of multi-type infections, including HPV 16. Some research suggests that multi-type infections, including HPV 16, do not increase carcinogenic risk compared with single-type infections [30]. Conversely, other studies have shown that multi-type HPV infections are associated with a significantly higher incidence of high-grade squamous intraepithelial lesions than single-type HPV infections [22]. Zhong et al. [25] suggested that these inconsistencies may be due to methodological differences, as specific HPV types exhibit distinct patterns of infection, highlighting the need for further research to confirm these findings.

Our study has the advantage of using a large population-based dataset of HPV test results from Korean women. Accumulating results from studies of the general population is as necessary as accumulating results from studies of specific patient populations and can be used to inform future cancer screening and vaccination recommendations.

Due to additional data collection limitations, we were unable to correlate the cervical health status of the participants; therefore, the association between specific HPV types and disease could not be determined. In addition, the data are based on HPV test results from a large Korean population and cannot be generalized to the entire Korean female population. Future studies are warranted to determine the relevance of interactions between specific types of HPV and disease expression.

## 5. Conclusions

HPV infections are prevalent among Korean women in their 20s and those aged 60 years and older. In addition, the most common multi-type infections were HPV 16 and 18 with HPV 52. The Korean public continues to lack a clear understanding of the HPV vaccine, which is attributed to a lack of understanding of HPV, infection, and vaccination [33]. While age and participant characteristics differ, the overall HPV infection rate (including duplicate infections) among 60,775 women from 2006 to 2011, prior to government support for national immunization, and among 16,669 women from 2020 to 2023, after government support, demonstrated similarities without a decrease [32]. Implementing preventive measures along with promotional strategies is required to enhance the understanding of HPV infections, including persistent infections and multi-type infections. Additionally, incentive policies should be enacted to increase vaccination rates among those eligible.

## Figures and Tables

**Table 1:** Comparison of positivity rates by age group (*n* = 16,669).

Category	Negative Rate	Positive Rate	χ^2^ (*p*-Value)	Single-Type Infection Rate	Multi-Type Infection Rate	χ^2^ (*p*-Value)
	*n* (%)	*n* (%)		*n* (%)	*n* (%)	
Total	10,555 (63.3)	6114 (36.7)	-	3572 (21.4)	2542 (15.3)	-
Age group						
20s	1109 (43.4)	1447 (56.6)	611.25 (<0.0001)	667 (26.1)	780 (30.5)	797.95 (<0.0001)
30s	2274 (64.6)	1244 (35.4)		733 (20.8)	511 (14.5)	
40s	3274 (71.7)	1295 (28.3)		869 (19.0)	426 (9.3)	
50s	2575 (67.1)	1260 (32.9)		805 (21.0)	455 (11.9)	
60s	1012 (59.5)	688 (40.5)		402 (23.6)	286 (16.8)	
70s+	311 (63.3)	180 (36.7)		96 (19.6)	84 (17.1)	
Age: Mean ± SD (range)	45.5 ± 12.1 (20–91)	42.9 ± 14.1 (20–86)		44.1 ± 13.3 (20–86)	41.4 ± 14.9 (20–85)	

**Table 2:** Age-specific positivity rates by number of multi-type infection viruses (*n* = 16,669).

Category	Negative Rate	Single-Type Positive Rate	Double-Type Positive Rate	Triple-Type Positive Rate	≥Quadruple-Type Positive Rate	χ^2^ (*p*-Value)
	*n* (%)	*n* (%)	*n* (%)	*n* (%)	*n* (%)	
Total	10,555 (63.3)	3572 (21.4)	1437 (8.6)	629 (3.8)	476 (2.9)	891.56 (<0.0001)
20s	1109 (43.4)	667 (26.1)	380 (14.9)	200 (7.8)	200 (7.8)	
30s	2274 (64.6)	733 (20.8)	310 (8.8)	130 (3.7)	71 (2.1)	
40s	3274 (71.7)	869 (19.0)	278 (6.1)	99 (2.2)	49 (1.0)	
50s	2575 (67.1)	805 (21.0)	261 (6.8)	117 (3.1)	77 (2.0)	
60s	1012 (59.5)	402 (23.6)	171 (10.1)	65 (3.8)	50 (3.0)	
70s+	311 (63.3)	96 (19.6)	37 (7.5)	18 (3.7)	29 (5.9)	

## Data Availability

The data used in this study were obtained from the U2Bio Laboratory. Due to company policies, the data cannot be made publicly available; however, access to the data is available from the corresponding author upon request.

## Supplementary Materials

The following supporting information can be downloaded at: https://www.mdpi.com/article/10.3390/pathogens14040369/s1, Table S1: Distribution of HPV infection types by age group, including single-type, high-risk (HR), low-risk (LR), and multi-type infections.

## Abbreviations

The following abbreviations are used in this manuscript:

DNA	Deoxyribonucleic acid
HPV	Human papillomavirus
HR	High-risk
RT-PCR	Real-time polymerase chain reaction
