# Supplementary material for: The Prevalence of Multi-Type Infections Among Human Papillomavirus Types in Korean Women
Source: Pathogens. 2025 Apr 9;14(4):369. doi: 10.3390/pathogens14040369 (PMC12030709; doi:10.3390/pathogens14040369)
Supplement: Supplementary file 1 [file pathogens-14-00369-s001.zip › pathogens-3517300-supplementary.pdf]

Supplementary Table S1.

Distribution of HPV infection types by age group, including single-type, high-risk (HR), low-risk (LR), and multi-type infections.

Age Group (n)	Total Positive Rate (%)	Single-Type (%)	High-Risk Single (%)	Low-Risk Single (%)	Multi-Type (%)
20s (n = 2,556)	56.61	26.10	10.99	15.10	30.52
30s (n = 3,518)	35.36	20.84	8.70	12.14	14.53
40s (n = 4,569)	28.34	19.02	6.85	12.17	9.32
50s (n = 3,835)	32.86	20.99	5.68	15.31	11.86
60s (n = 1,700)	40.47	23.65	6.71	16.94	16.82
70s+ (n = 491)	36.66	19.55	7.33	12.22	17.11

*Note: “High-Risk” and “Low-Risk” refer to HPV types categorized according to their oncogenic potential. “Multi-Type” includes infections involving two or more HPV types.
